# Comparative effectiveness of endoscopic and microscopic adenoma removal in acromegaly

**DOI:** 10.3389/fendo.2023.1128345

**Published:** 2023-09-11

**Authors:** Natalya Vassilyeva, Natmir Mena, Kiril Kirov, Ekaterina Diatlova

**Affiliations:** ^1^ Department Emergency Medicine, Anesthesiology and Resuscitation, Karaganda Medical University, Karaganda, Kazakhstan; ^2^ Department of General Surgery, Medical University Pleven, Pleven, Bulgaria; ^3^ Research Institute, Medical University Pleven, Pleven, Bulgaria; ^4^ Department of Nursing Management and Social Work, Sechenov First Moscow State Medical University, Moscow, Russia

**Keywords:** somatotropinoma, acromegaly, adenomectomy, microscopic techniques, endoscopic techniques

## Abstract

**Introduction:**

Somatotropinomas are the main cause of acromegaly. Surgery is the primary and most efficient method of treatment. The study aimed to compare the radicality of small-sized and medium (<30 mm) somatotropinoma removal and the incidence of postoperative complications in patients with acromegaly when using microscopic and endoscopic techniques.

**Methods:**

In this randomized controlled trial, a total of 83 patients with acromegaly underwent transspheroidal endoscopy or microscopic surgery. Somatotropinoma was the cause of acromegaly in all cases. Patients were randomly divided into two comparison groups depending on the applied surgical technique. Group 1 (n = 40) consisted of patients who underwent adenomectomy with transnasal transsphenoidal access by a microscope. Group 2 (n = 43) included patients who underwent the same surgical procedure with an endoscope. The following indicators were assessed: radicality of tumor removal, treatment effectiveness, postoperative complications, and remission rate.

**Results:**

The study has shown that removal of somatotropinoma in patients with acromegaly using endoscopic technique increases the radicality of tumor removal in comparison with microscopic technique. Total removal of somatotropinoma was successful in 88.4% of cases when using the endoscopic technique. Secondly, the segmentation of patients according to their tumor characteristics poses challenges, primarily owing to the rarity of acromegaly as a disease. The difference between groups was not statistically significant (p=1.02). There were no statistically significant differences in basal GH level and IGF-1 level between groups (p=0.546 and p=0.784, respectively).

**Discussion:**

Endonasal transsphenoidal endoscopic adenomectomy is proven efficacy, a less traumatic degree, and higher somatotropinoma removal radicality. Both surgical methods lead to disease remission.

## 1 Introduction

Somatotropinomas are the main cause of acromegaly, a chronic progressive disease associated with hypersecretion of growth hormone (GH) and insulin-like growth factor 1 (IGF-1) (1, 2).

Acromegaly is a rare disease (orphan disease) which affects women and men equally often (3). The average age of acromegaly diagnosis is 45.2 years (4). The worldwide prevalence of acromegaly varies from 28 to 137 cases per 1 million population, depending on the country (5).

In Bulgaria, for example, the prevalence of acromegaly is 49 cases per 1 million (6); and in Russia, it is 30 cases per 1 million (7). However, many episodes of the disease remain undiagnosed and data from national registries are incomplete (8, 9).

Changes in the musculoskeletal system at acromegaly are associated with the GH and IGF-1 levels, playing a vital role in the regulation of cartilaginous and osseous tissue homeostasis, bone growth in length, and the increase in the mass of osseous tissues (10, 11). Osteoarthropathy occurs in nearly 70% of patients suffering from acromegaly. Acromegaly in all inner organs facilitates active processes of hypertrophy and hyperplasia and causes metabolic complications: insulin resistance and hyperglycemia. In most cases, these complications lead to diabetes mellitus and hyperlipidemia (10, 12). In addition, the disease causes many other complications - heart failure, arterial hypertension, arthropathy, thyroid dysfunction, cephalalgia, and a higher incidence of neoplasms (13, 14).

Mortality without adequate treatment is 2.0 to 4.0 times greater than in the general population (15). The longer the disease lasts and the greater the GH, the shorter a patient’s life expectancy (16, 17).

Given the seriousness of the disease and the high percentage of systemic complications, deteriorating a patient’s life quality, timely and correct treatment of acromegaly is significantly crucial (18, 19). The main components of acromegaly therapy include the following: tumor resection or stabilization of its size, a persistent and steady decrease of GH and IGF-1 concentration, preservation of functional activity of pituitary gland, reduction of clinical symptoms of the disease, and the prevention of acromegaly recurrence (20, 21).

Surgery is the primary and most efficient method of treatment. It can result in a stable and rapid remission of acromegaly (22–24). Transcranial and transnasal operations on the pituitary gland of the acromegaly have long been performed using a microscope (22).

In recent years, transcranial access has been used infrequently. Most somatotropins are removed through transsphenoidal access when the tumor is small or medium in size. The endoscopic technique development has made it possible to endonasal transsphenoidal somatotropinoma removal with an endoscope. This technique reduces the risk and increases the radicality of the surgical procedure (21, 25, 26). Sometimes, using two techniques simultaneously (endoscopic assistance) is recommended. That is, surgical access for somatotropinoma removal and the correction of plastic defects are performed under a microscope. In this case, an endoscope is required to control the removal of tumor parts in hard-to-reach locations (22). The efficacy and advantage of endoscopic technique over microscopic technique are undeniable and proven in large somatotropinomas (25). However, the question of the effectiveness and feasibility of the endoscopic technique in medium- and small-sized neoplasms remains relevant and needs further investigation. This is important as somatotropinomas are rarely large and, in most cases, easily accessible using a microscope by the classic transsphenoidal technique.

The study aimed to compare the radicality of small-sized and medium (<30 mm) somatotropinoma removal and the incidence of postoperative complications in patients with acromegaly when using microscopic and endoscopic techniques.

## 2 Material and methods

### 2.1 Patients and study design

This study is a randomized controlled trial examining 154 patients with acromegaly. A total of 83 eligible patients underwent transspheroidal endoscopy and microscopic surgery. The interventions took place in the neurosurgical clinic of the Dr. Georgi Stranski University Hospital (Pleven, Bulgaria) within the period between 2017 and 2021. Of those operated, 50 (59.0%) were female, and 33 (41.0%) were male. The age of the study participants varied between 23 and 66 years (43.75 ± 9.23). All patients underwent primary operations. Somatotropinoma was the cause of acromegaly in all cases.

The present study uses the Classification of Pituitary Adenomas developed by the Burdenko Neurosurgical Institute in Moscow, Russia. The Russian Association of Neurosurgeons recommends the mentioned Classification (27).

Patients were randomly divided into two comparison groups depending on the applied surgical technique. The randomization process was conducted using a computer program by a specialist who was not aware of the study design. Patients were numbered from 1 to 83 by their registration sequence upon arrival for treatment. The surgical technique used was determined during the preparation of the patient for the procedure. When distributing patients into groups, their gender, age, tumor size, the direction of tumor growth, and degree of invasion were taken into account.

Thus, Group 1 (n = 40) consisted of patients who underwent adenomectomy with transnasal transsphenoidal access by a microscope. Group 2 (n = 43) included patients who underwent the same surgical procedure with an endoscope. The two groups were then compared to determine which surgical method was more effective. There were no statistically significant differences between groups by sex, age, tumor size, tumor growth direction, and the invasion (P> 0.05, Mann-Whitney U-test).

The inclusion criteria for the study were: 1) patients aged 18 years or older; 2) acromegaly diagnosis; 3) the evidence of microsomatotropinoma (<15 mm), small-sized somatotropinoma (16 to 25 mm) or medium somatotropinoma (26 to 35 mm); and 4) signed informed consent to participate in the study.

The exclusion criteria were: 1) patients aged under 18 years; 2) patients with large or giant somatotropinoma (>35 mm); 3) laterosellar expansion of the adenoma with secondary non-capsular nodes, a small sella turcica, or a narrow entrance to the secondary nodes of the tumor; 4) refusal to undergo surgical intervention for acromegaly; 4) patients diagnosed with other endocrine disorders that may affect the outcome; 5) acute myocardial infarction earlier than 3 months prior to the study; 6) acute cerebral circulation disorder earlier than 3 months prior to the study; 7) decompensated concomitant pathology; 8) mental illness; 9) refusal to participate in the study.

**Figure 1:**
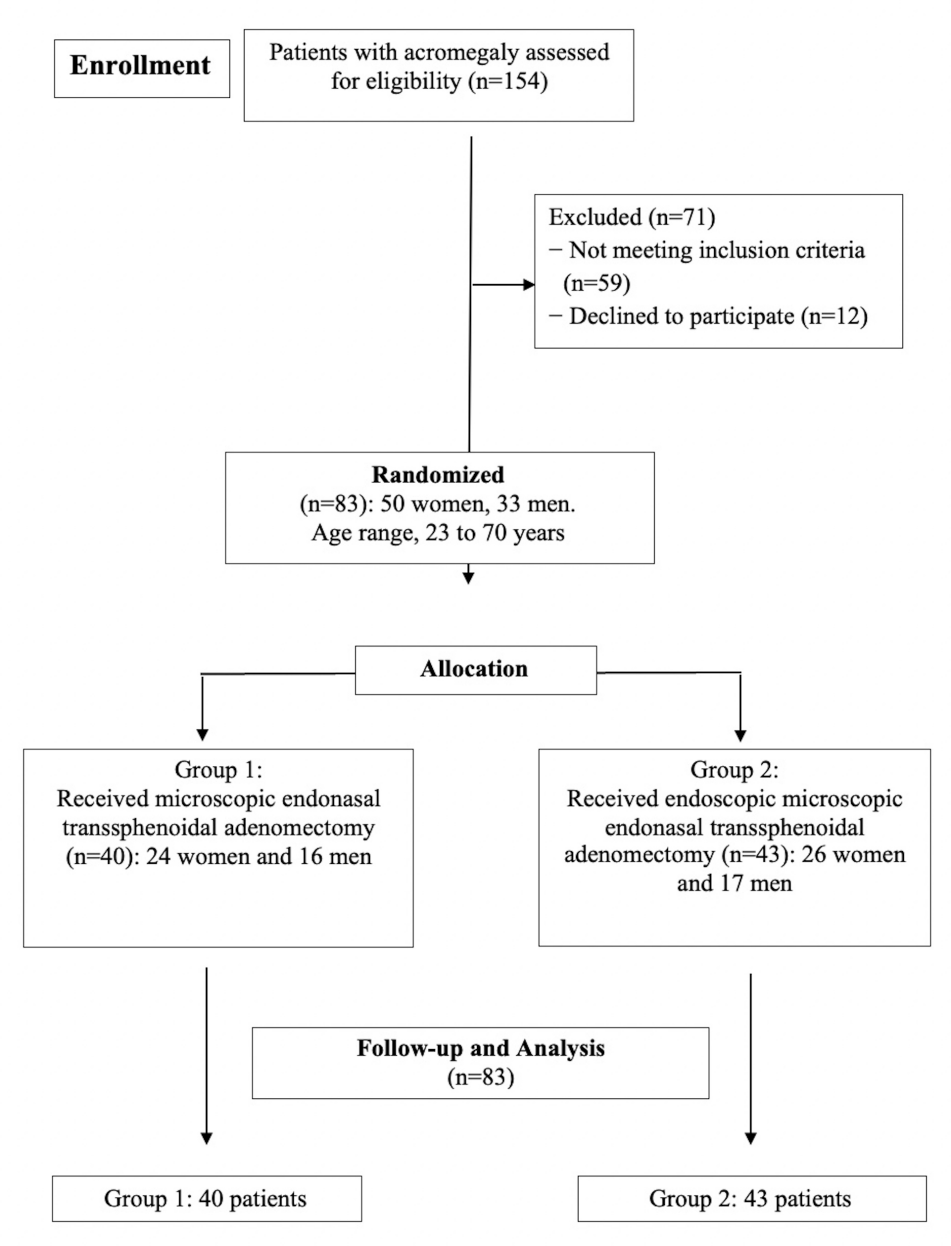
Study design.

Patients were observed postoperatively during hospital stay and within one month after their discharge. All data obtained during the study were kept in a database and processed after all patients underwent the necessary procedures. **Figure 1** shows the flowchart of the study.

### 2.2 Clinical, laboratory and instrumental examination

The diagnosis of acromegaly was verified following the recommendations of *A Pituitary Society* (28) and *An Endocrine Society Clinical Practice Guideline* (17) based on the analysis of clinical symptoms and disease syndromes, laboratory tests (determination of GH, IGF-1 in blood, glucose tolerance test), MRI and histological examination of tumor after surgical intervention. Several laboratory tests were required for all patients in the study to identify a concomitant pathology. These included urinalysis, general blood tests, and biochemicals (glucose, urea, creatinine, total bilirubin, alanine and aspartat aminotransferase, ionogram, and lipidogram). The GH and IGF-1 levels were estimated on an empty stomach (after fasting for at least 8 hours) by enzyme immunoassay (ELISA) using standard ELISA kits (DRG Diagnostics) on a StatFax 2100 automatic analyzer (Awareness Technology Inc., USA). In the absence of diabetes, the patients completed a blood glucose test (75 g) to determine blood GH suppression at 0, 30, 60, 90 and 120 minutes. The study of this parameter was carried out by the glucose-oxidant method on a BiosenC-line analyser (EKF diagnostic, Germany).

Craniographic and lateral sinus radiograph were performed in the patients using a GE Proteus XR/a system (Germany) to detect changes in the sella turcica features of pituitary adenoma (i.e., size, shape, the presence of anterior osteoporosis, and adjacent bone structures). All patients who had no contraindications for being in a magnetic field underwent an MRI of the head using the Optima MR450w 1.5T GEM device (GE, USA) to study the characteristics of the tumor (size, structure, etc.). In case of contraindications, CT of the head was performed using the Cytom 16 device (STERNMED, Germany).

### 2.3 Neurosurgical treatment

Indications for pituitary adenoma surgery were active adenoma growth, visual impairment or other neurological defects, and endocrine syndromes that require medical treatment. The operations were performed by a single team of surgeons trained in microscopic and endoscopic techniques and with significant practical experience in both techniques. The experience of surgeons with microscopic and endoscopic techniques in the present study was comparable. A team of surgeons performed more than 50 operations for each method in the year preceding the study. Preoperative therapy with somatostatin analogues was not carried out because there is currently no evidence that preoperative preparation with somatostatin analogues improves surgical outcomes in patients with somatotropinomas.

Adenomectomy was performed using a microsurgical technique with a surgical endoscope or microscope. Group 1 received microscopic transsphenoidal surgery with a Leica M695 D2 surgical microscope (Leica, Switzerland) via the Hirsch approach. The patient was placed in a semi-sitting position, and the lateral skull base images were obtained using an electron-optical converter (EOP) installed perpendicular to the sagittal projection of the skull. Before surgery, patients underwent either the lumbar catheter placement or puncture by air (3-5 ml) to highlight the suprasellar region and basal cisterns.

The main stages of microscopic endonasal transsphenoidal intervention were as follows:

1) After the nasal mucosa was treated with an antiseptic solution, a nasal speculum was installed.
2) Subsequently, the mucous membrane and the posterior segment of the nasal septum underwent dissected.
3) The mucous membrane was separated from the anterior wall of the sphenoid sinus.
4) Using bone cutters or a high-speed burr, two holes were sequentially made in the anterior wall of the sphenoid sinus and on the bottom of the sella turcica.
5) The dura mater was dissected, and the tumor was removed in a strictly defined sequence: first, the endosellar and infrasellar parts were cut out, and then the suprasellar and laterosellar parts were removed. The instrument manipulations in the area with limited visual control were controlled through a microscope using the EOP. Thus, it was possible to assess the prevalence of the suprasellar region tumors and their removal radicality (the site of the removed tumor becomes lighter on the transducer screen).
6) After the tumor removal, the opened bottom of the sella turcica was closed (suturing of the dura mater, placement of the nasal septum fragments between the bottom of the saddle and the dura mater, sealing with a Tachocomb plate, etc.).
7) Finally, the tamponade of the nasal cavity was carried out with air ducts placed in the nasal passages and a sling bandage applied to the nose.

Group 2 received endoscopic endonasal transsphenoidal surgery by rigid 4 mm endoscopes (Karl Storz, Germany) with four viewing angles (0°, 30°, 45° and 70°). A patient was lying on the back with their head raised 15-30°. Immediately before the operation, tampons moistened with vasoconstrictor drugs were placed inside the patient’s nasal cavity.

The main stages of endoscopic endonasal transsphenoidal intervention were as follows:

1) After the general examination of the nasal cavity and the use of the antiseptic solution, the nasal passage was selected for the nasal septum curvature.
2) Increasing the additional lumen of the operating channel required the middle turbinate lateralization. A nasal dilator was placed in cases of a deviated nasal septum or a narrow lumen of the nasal passages.
3) After discovering the natural fistula of the main sinus, partial removal of the nasal mucosa from the anterior wall of the sphenoid sinus was carried out via coagulation and using a debrider.
4) After removing the mucosa, a hole was made in the anterior wall of the main sinus by expanding its mouth with the lateral displacement the nasal septum posterior parts.
5) The sphenoid sinus mucous membrane coagulated in the area of trepanation.
6) After making a hole in the sella turcica bottom, the dura mater was dissected and the tumor tissue was removed.
7) Upon tumor resection, the floor of the sella turcica was securely sealed.

Compared to the microscopic intervention, the endoscopic procedure did not require the tamponade of the nasal cavity. Therefore, the final step was the repositioning of nasal turbinates. In case of bleeding, a hemostatic nasal sponge can be inserted into the middle nasal passage.

In all cases where pituitary adenoma was removed to prevent postoperative liquorrhea (even in the absence thereof), the sella turcica was sealed using TachoComb Baxter plates in combination with fibrin-thrombin glue (Evisel ETICON). In case of large defects at the saddle bottom, a temporary balloon catheter was placed into the main sinus cavity.

### 2.4 Evaluation of treatment outcomes and postoperative complications

The study of the level of basal GH was carried out on the 1st day after the operation. GH level <2 ng/mL may be a predictor of long-term remission, but the value of this test is limited due to the possible increase in the level of GH as a result of excessive production by normal pituitary somatotrophs as a result of surgical stress on the organism. After surgery for 2 weeks, monitoring of electrolyte levels, symptoms of diabetes insipidus and syndrome of inappropriate secretion of antidiuretic hormone, monitoring of adrenal function was performed. The results of surgical treatment were evaluated 12 weeks after the operation. The tumor removal radicality was evaluated based on the operating surgeon’s subjective opinion, clinical data, postoperative hormonal findings, and postoperative MRI data or CT data in the presence of contraindications to stay in a magnetic field. Total tumor removal refers to the surgical outcome where residual tumor is found neither according to the surgeon’s opinion nor according to the postoperative CT and MRI data. In the subtotal tumor removal, the residual tumor volume did not exceed 20% of the original tumor. The postoperative CT and MRI analysis provided no evidence of residual tumor. At the same time, evidence of the blood hormone level returning to normal was absent. Those cases were classified as subtotal resection. The tumor removal was classified as partial when less than 80% of the original adenoma was excised. The evaluation of the MRI and CT scans was performed by two independent radiologists unfamiliar with the study design.

The level of IGF-1 and GH was studied during an oral glucose tolerance test (75 g of glucose per os with a study of the level of GH every 30 minutes for 2 hours). If the level of IGF-1 in the blood decreased but did not reach the norm, the level of IGF-1 was re-examined after 9–12 weeks due to the possible delayed normalization of its level in some patients.

Treatment effectiveness was evaluated by analyzing the dynamics of the primary syndromes: endocrine, visual, and neurological. The complications were classified as mild, moderate, and severe. Mild complications refer to the new symptoms emergence associated with the underlying or concomitant disease, which slightly worsens the patient’s condition and ability to work. Nevertheless, the impact on health and working ability is not critical though. Moderate complications make patients unable to work but they can still take care of themselves. Severe complications imply that a patient loses their ability to work and self-care. In this case, they require outside help.

### 2.5 Evaluation of disease remission in patients with acromegaly

In most cases, after surgery, IGF-1 normalizes within 1 month, and the level of GH during the first two weeks, however, in some patients, delayed normalization of their levels is possible. Therefore, the postoperative period of 6 months was taken as the minimum period for assessing remission.

The analysis at 6- and 12-month follow-ups and involved 21 patients after microscopic surgery and 23 patients after endoscopic surgery. The remission criteria for the follow-up of patients with acromegaly were the absence of the disease’s clinical signs, a basal GH level of <2.5 ng/mL, a minimum GH level of <1 ng/mL (2.7 mU/L) in the oral glucose tolerance test, and IGF-1 level within the normal range according to gender and age. The basal GH level should not have been lower than 6.7 mU/L. The minimum level of GH had to be not lower than 2.7 mU/L in the oral glucose tolerance test after its intake when measuring the indicator every 30 minutes for 2 hours (29). The concept of remission included only those patients who had normalization of hormonal parameters according to all three criteria: the basal level of GH, GH in oral glucose tolerance test and IGF-1.

### 2.6 Statistical analysis

Statistica 6.1 (StatSoft, Inc., USA) was the software for statistical data analysis in this study. Means and standard deviations (M+s; normal distribution) or medians and quartiles (Me [Qi; Q3]; non-normal distribution), and absolute and relative frequencies and 95% confidence intervals (CI) represent descriptive statistics of quantitative traits. The Shapiro-Wilk test was a tool to assess the normality of quantitative variables. Nonparametric ANOVA (Kruskal-Wallis and Mann-Whitney U tests) compared unrelated groups by quantitative and ordinal characteristics. The study involved multiple *post hoc* comparisons according to Siegel and Castellan. The Wilcoxon test compared related groups. The qualitative comparisons between unrelated groups resulted from using the Chi-square test, Spearman’s T-test and Fisher’s exact test. The results were considered statistically significant at P<0.05.

### 2.7 Adherence to ethical norms

The research corresponds to international principles and standards of biomedical ethics: International Ethical Guidelines for Biomedical Research Involving Human Subjects of the Council for International Organization of Medical Sciences, Rules of ethical principles for scientific medical research with human participants”, approved by the Declaration of Helsinki (1964-2013), ICH GCP principles (1996), Council Directive 609 (of 24.11.1986), the Convention on Human Rights of the Council of Europe.

Only patients who signed a voluntary informed consent form to participate in this study were enrolled. The participants received complete answers to all the raised questions. The focus was on interpreting treatment methods, their advantages and disadvantages, consequences, potential complications, etc. The patients were aware that they had the right to withdraw at any stage of the study. The patient was provided with copy of a signed informed consent form to participate in the study. The Ethical Committee of the Medical University Pleven, Bulgaria reviewed and approved the study design (protocol 117-a dated 12/16/2016).

## 3 Results

### 3.1 Demographic and clinical profiles

**Table 1** presents the demographic characteristics of patients. In the context of the study, the female representation among the participants in groups 1 and 2 amounted to 60% and 60.5%, respectively. The mean age of patients was 44.12 ± 9.14 and 43.26 ± 8.64 years, respectively. The dominant age groups were patients aged 41 to 50 (37.5% and 41.9%) and 51 to 60 years of age (35% and 27.9%). There were no statistically significant differences between the comparison groups. Time to diagnosis after the onset of first symptoms (changes in limb size, swelling, and facial features) averaged to 7.5 years (3-22 years). The mean time from diagnosis to surgical treatment was 2.5 years (2-16 years).

**Table 1:** Demographic profile of patients and their tumor characteristics.

	Group 1 (microscope technique, n = 40)	Group 2 (endoscopic technique, n = 43)	P value
Gender			
Men	16 (40%)	17 (39.5%)	Р=0.598
Women	24 (60%)	26 (60.5%)	
Age groups			
Average age	44.12 ± 9.14	43.26 ± 8.64	Р=0.435
21 to 30 years	2 (5%)	1 (2.3%)	Р=0.451
31 to 40 years	5 (12.5%)	7 (16.3%)	
41 to 50 years	15 (37.5%)	18 (41.9%)	
51 to 60 years	14 (35%)	12 (27.9%)	
61 to 70 years	4 (10%)	5 (11.6%)	
Tumor sizes			
Microsomatotropinomas (<10 mm)	3 (7.5%)	4 (9.3%)	Р=0.867
Small-sized somatotropinomas (11 to 20 mm)	25 (62.5%)	28 (65.1%)	
Medium-sized somatotropinomas (21 to 30 mm)	12 (30%)	11 (25.6%)	
Samatotropinomas localization			
Endosellar	17 (42.5%)	21 (48.8%)	Р=0.312
Endoextrasellar (multidirection extension is possible), including:	23 (57.5%)	22 (51.2%)	
Suprasellar	19 (47.5%)	21 (48.8%)	
Retrosellar	0 (0%)	1 (2.3%)	
Anthesellar	1 (2.3%)	1 (2.3%)	
Infrasellar	12 (30%)	14 (32.6%)	
Laterosellar	7 (17.5%)	10 (23.6%)	
Invasion degree of endoextrasellar somatotropinomas			
Moderate invasion	21 (91.3%)	19 (90.5%)	Р=0.183
Severe invasion (extension in more than 2 directions)	2 (8.7%)	3 (9.5%)	

Of all patients, 73 (88%) had some somatic pathology before surgery. There were 46 mild patients (63%), 23 moderate patients (31.5%) and 4 severe patients (5.5%).

**Table 1** shows the tumor characteristics. In the present study, most patients (63.9%) had small-sized somatotropinomas (11 to 20 mm). Endoextrasellar tumor localization (tumors spread outside sella turcica) comprised 54.2% of cases, and other cases involved endosellar localization (tumors that do not extend beyond sella turcica). The majority of patients (89%) with endoextrasellar tumor localization experienced suprasellar tumor extension, sometimes combined with infrasellar or laterosellar extensions. Most patients (89%) had moderate tumor invasion (no more than 2 growth directions). Other patients demonstrated a pronounced tumor invasion (tumor extension in more than two directions). There were no statistically significant differences between the comparison groups.

### 3.2 Treatment outcomes

**Table 2** shows the anesthesia time, surgery time, and postoperative days of hospital stay. Significantly, the duration of anesthetic management may be influenced not only by the time required to induce anesthesia but also by the intubation procedure. Some patients experienced serious changes in the nasopharynx. The changes required the surgeon to use endoscopic equipment to place an endotracheal tube. The denser tumors took more time to remove. In general, however, the average anesthesia time and the average surgery time were shorter during endoscopic operations, and this difference was statistically significant (P <0.001, Mann-Whitney U-test). Meantime, the postoperative duration of hospital stay after microscopic intervention was statistically longer than after endoscopic surgery (P<0.001, Mann-Whitney U-test).

**Table 2:** Anesthesia time, surgery time, and postoperative days of hospital stay.

	Group 1 (microscope technique, n = 40)	Group 2 (endoscopic technique, n = 43)	P value
Anesthesia time	237 ± 68 min (107 to 564 min)	202 ± 77 min (95 to 440 min)	Р<0.001
Surgery time	176 ± 56 min (64 to 355 min)	142 ± 54 min (52 to 312 min)	Р<0.001
Postoperative days of hospital stay	7 ± 1.4	5 ± 1.4	Р<0.001

When analyzing the radicality degree of adenomectomies depending on the surgical technique, a complete tumor removal was possible 1.4 times (p<0.05) more frequent in adenomectomy with the endoscopic technique compared to the microscopic one (**Table 3**). The sub-total tumor removal was performed significantly more often (3.6 times, p<0.05) using microscopic technique.

**Table 3:** Volume comparison of surgical removal of somatotropinomas using a microscope and endoscope in patients with acromegaly.

Scope of operation	Group 1 (microscope technique, n=40)	Group 2 (endoscopic technique, n=43)	OR	95% CI
Partial	3 (7.5%)	1 (2.3%)	4.40	0.38-50.82
Subtotal	10 (25%)	4 (9.3%)	4.55*	1.20-17.24
Total	27 (67.5%)	38 (88.4%)	5.10*	1.51-17.24

*the difference is statistically significant compared to the group operated with the microscope.

After adenomectomy, patients in both groups showed a tendency to improve their somatic status (better general well-being, normalized arterial pressure, reduced oedema, and sweating): 19 (48.1%) patients in the microscopic adenomectomy group and 25 (57.1%) patients in the endoscopic adenomectomy group. The difference between groups was not statistically significant (OR=1.23, 95% CI [0.57–3.61], p>0.05). In addition, some patients noticed an improvement in neurological status (reduced severity of paresthesias and headaches and better emotional state): 17 (42.5%) patients in the microscopic group and 21 (42.5%) patients in the endoscopic group. The difference between groups was not statistically significant (OR=1.16, 95% CI [0.46–2.90], p>0.05). After the microscopic removal of the tumor, improvement in visual functions was observed in 10 (25.9%) patients, and after endoscopic removal, it was observed in 22 (51.8%) patients. The difference between groups was statistically significant (OR=3.07, 95% CI [1.12–8.41], p<0.05).

As for the complications of surgical treatment, intraoperative liquorrhea was present in 21 (51.9%) patients operated on using a microscope and in 15 (35.7%) patients operated on using endoscopy. The intergroup difference was statistically significant (OR=3.02, 95% CI [1.21-7.50], p<0.05). One (3.7%) patient in group 1 and one (2.3%) patient in group 2 experienced a decrease in visual acuity (OR=2.12, 95% CI [0.13-35.17], p> 0.05). One microscopic patient (3.7%) demonstrated hemorrhagic complications (in the form of an acute hemorrhagic disorder of the brain circulation). One patient (3.7%) in group 1 and two (4.6%) patients in group 2 reported an increase in headache intensity postoperatively (OR=1.04, 95% CI [0.09–11.98], p>0.05). Postoperative epistaxis was observed in 2 patients in the endoscopic technique group and was absent in patients in the microscopic technique group. There were no cases of postoperative meningitis or development of sinusitis in the long term. Importantly, there was not a single case of lethal results in endoscopic and microscopic procedures for tumor removal. In some cases, the patients had endocrine complications following the tumor removal (**Table 4**), namely, diabetes insipidus, hypocortisolismus, and hypothyroidism.

**Table 4:** The comparison of Postoperative Incidence of Endocrine Disorders in Patients with Acromegaly with Microscopic and Endoscopic Methods of Tumor Removal.

Endocrine disorder	Group 1 (microscope technique, n=40)	Group 2 (endoscopic technique, n=43)	OR	95% CI
Hypocortisolismus	2 (5%)	4 (9,3%)	2.55	0.28-22.97
Diabetes insipidus	6 (15%)	8 (18,6%)	1.25	0.35-4.42
Hypothyroidism	2 (5%)	3 (7%)	1.47	0.15-14.85

The incidence of endocrine disorders varied depending on adenomectomy technique. Diabetes insipidus, hypocortisolismus, and hypothyroidism occurred more frequently after endoscopic rather than microscopic surgery. However, this difference is not statistically significant (p>0.05).

In general, the complications following endoscopic intervention were mainly mild. Moderate and severe complications occurred less frequently compared to microscopic operations.

### 3.3 Evaluation of disease remission

In the follow-up groups, a small proportion of patients achieved remission immediately after surgery – 14% in group 1 and 21% in group 2. At 6-month follow-up, remission was diagnosed in 36% of patients in group 1 and 41% in group 2. At the 12-month follow-up, 44% of group 1 and 48% of group 2 managed to achieve remission. The difference between groups is not statistically significant (p=1.02, at 12-month follow-up).

After 12 months, the basal GH level normalized in 68% of patients in group 1 and 72% in group 2. By this follow-up, the GH level in the oral glucose tolerance test reached 65 and 70%, respectively. IGF-1 normalized in 56 and 61% of patients, respectively. There was no statistically significant difference in these two indicators was found between groups (p=0.546 and p=0.784, respectively). Thus, both surgical methods lead to disease remission, and the effects are generally similar.

## 4 Discussion

This research endeavor undertook a comparative investigation of two distinct adenomectomy techniques in the context of acromegaly patients: transnasal transsphenoidal adenomectomy employing a traditional microscope versus utilizing an endoscope. According to the results of the study, it was found that the endoscopic technique increases the radicality of tumor removal in comparison with microscope one. For example, total elimination of samatotropinoma was performed in 88.4% of endoscopic patients and 67.5% of microscopic patients (OR=5.10, 95% CI [1.51–17.24], p<0.05). When using a microscope, the percentage of subtotal tumor removal was relatively high (25%). However, it was significantly lower (9.3%) in endoscopic cases (OR=4.55, 95% CI [1.20–17.24], p<0.05). Such advantages of the endoscope over the microscope are associated with better illumination of an operating field during endoscopic operations, better angle of vision, increased handling capabilities, etc.

In this study, anesthesia time during endoscopic operations was shorter than microscopic procedures (202 ± 77 minutes versus 237 ± 68 minutes, Р<0.001). Endoscopic procedures were also shorter (142 ± 54 minutes versus 176 ± 56 minutes, Р<0.001). However, the operation time strongly depends on a tumor’s density, size, and localization; therefore, there was a considerable difference in the operating time in both the endoscopic (52 to 312 min) and microscopic (64 to 355 min) groups. Russian neurosurgeons reported that on average, endoscopic operations take 25-30 minutes less time (30), which is generally consistent with our data. Notably, it takes significantly longer to sit a patient before surgery and lay them down after awakening during microscopic operations. In addition, Prajapati et al. have reported shorter operating times in endoscopic surgery (31). In our opinion, using an endoscope with a large viewing angle, better illumination of the operating area, and greater manipulative capabilities can reduce the operating time. Nevertheless, considering the myriad of factors influencing operating time, it would not be appropriate to categorize it as either a disadvantage or an advantage of these surgical techniques.

The number of patients with improved visual function following surgical treatment also confirmed the increased efficiency of endoscopic adenomectomy for acromegaly. In particular, the endoscopic technique improved eyesight in 51.8% of patients, while the microscopic procedure had the same effect in 25.9%. The intergroup difference was statistically significant (OR=3.07, 95% CI [1.12–8.41], p < 0.05). It can be due to the tumor removing radicality that presses on the area of the sella turcica stool (compressing the optical nerves) when using the endoscopic technique.

In the postoperative period, mild complications prevailed when using the endoscopic technique of tumor removal compared to the microscopic technique Most of them were hormonal (diabetes insipidus, hypocortisolismus, hypothyroidism), but there was no statistically significant difference among the groups in the frequency of these complications.

However, intraoperative liquorrhea was significantly higher with the microscope compared to the endoscope: 51.9% vs. 35.7% (OR=3.02, 95% CI [1.21–7.50], p<0.05). Intraoperative liquorrhea is common in endoscopic surgery, but this complication can also occur after microscopic operations. It is more often detected in cases of more radical tumor removal during endoscopic operations. Broersen et al. (32) have reported a higher rate of CSF leaks after endoscopic transsphenoidal surgery. At the same time, Chen et al. (33) have found no difference in CSF leak between endoscopic and microscopic groups. Castaño-Leon et al. (34) have reported fewer CSF leak rates with endoscopic surgery than with microscopic procedures.

It is worth noting that the incidence of intraoperative liquorrhea depends rather on the direction of tumor growth, its size, and the sella turcica diaphragm structure than on the illumination quality of the operating wound. However, when working with the suprasellar part of the tumor, it is vital to achieve the best visual control possible under endoscopic illumination because it allows the detection of even the small CSF fistulas and successfully prevents the increase in CSF leak rates.

The overall disease remission evaluation revealed that the studied approaches had no clear advantages over one another. However, patients had a slightly higher remission rate after endoscopic adenomectomy (44% vs. 48%, respectively, P = 1.000). The results obtained in this study are consistent with those of other studies on the issue. For example, in a study by Fathalla et al. (24) in Canada, the total resection of the tumor was significantly higher (61% compared to. 42%, p = 0.05) in the group of patients who underwent endoscopic versus microscopic adenomectome. Furthermore, there was a trend towards a higher total resection rate in the presence of a cavernous sinus (48% vs. 14.2%, p = 0.09).

Another study involved 113 patients with acromegaly who underwent surgical removal of the adenoma (endoscopic – 66 patients, microscopic – 47 patients). This study reported more frequent cases of diabetes insipidus development after the endoscopic (9.1% vs. 4.3% of cases, p = 0.466). At the same time, microscopic treatment resulted in a higher incidence of hypothyroidism (in 21.2% of cases in microscopic compared to 12.2% in endoscopic procedures, p < 0.001) (26).

A recent meta-analysis of 21 studies (involving 292 patients with acromegaly who underwent endoscopic adenomectomy and 648 patients who underwent microscopic adenomectomy) showed that endoscopic technique was more likely to result in total tumor resection compared to microscopic one (53.5% vs. 46.6%, respectively). In addition, vision improvement occurred in 73.2% and 49.6% of patients in endoscopic and microscopic groups (23).

Some studies note that the microscopic transsphenoidal approach provides a limited view due to the narrow corridor to the Turkish saddle. The endoscopic methods, on the other hand, offer a wider view of the medial and inferior walls of the cavernous sinus, enabling a complete tumor resection and, consequently, hormonal remission (35, 36).

Thus, applying endoscopes with different viewing angles can significantly facilitate examining the surgical intervention area in good light conditions. The ability to remove the tumor under direct visual control increases the operation radicality and reduces the risk of damaging vital anatomical structures. Another advantage of endoscopic interventions is the safer placement of a nasal port in a narrow or curved nasal passage. The presence of postoperative nasal tamponade several days after the surgery increases (twofold on average) the duration of hospital stay. Therefore, the endoscopic technique is safer since it eliminates the need for postoperative nasal tamponade and reduces postoperative pain.

At the same time, microscopic somatotropin removal is a completely reliable and proven method. If the hospital has equipment for microscopic operations only, there is no fundamental need to purchase additional endoscopic equipment to enable operations in the chiasmal-sellar regions. However, patients with large tumors and significant extracellular extension require treatment in specialized centers.

The present study has several limitations. First, the tumor removal radicality and procedure time depend on the tumor size, the invasion direction and degree, and the tumor density. The present study was limited to tumors <30 mm in size, located within the sella turcica and extending moderately in multiple directions. Secondly, the segmentation of patients according to their tumor characteristics poses challenges, primarily owing to the rarity of acromegaly as a disease. This problem was solved by differentiating tumor sizes and extension directions in comparison groups. In addition, there were not enough patients with dense tumors in the sample to draw any conclusions.

## 5 Conclusion

The study has shown that endoscopic surgery’s average anesthesia and procedure times are significantly shorter than those of microscopic surgery. Compared to the microscopic technique, the endoscopic technique increases the radicality of somatotropinoma total removal in patients with acromegaly. In general, the complications following endoscopic intervention were mainly mild. Moderate and severe complications occurred less frequently compared to microscopic operations. Since most complications are hormonal and often regress during the late postoperative period, the endoscopic intervention seems to have less severe consequences. The exclusion of unnecessary postoperative nasal tamponade during endoscopic surgery reduced the length of hospital stay, the frequency of moderate and severe postoperative complications, and pain, leading to rapid postoperative recovery. In general, both microscopic and endoscopic procedures show similar effectiveness in achieving disease remission. Given the above advantages, endoscopic endonasal transsphenoidal adenomectomy is the preferred method for surgical treatment of acromegaly. This method has proven efficacy, lower traumatic effect, and a higher degree of somatotropinomas removal radicality. At the same time, the microscopic somatotropin removal technique is reliable as well. It can be applied to remove chiasmal-sellar region tumors, when there is no required equipment or skilled personnel to perform endoscopic endonasal transsphenoidal adenomectomy.

## Data availability statement

The original contributions presented in the study are included in the article/supplementary material. Further inquiries can be directed to the corresponding author.

## Ethics statement

The authors declare that the work is written with due consideration of ethical standards. The studies involving humans were approved by Medical University Pleven, Bulgaria (protocol 117-a dated 12/16/2016). The studies were conducted in accordance with the local legislation and institutional requirements. Informed consent was obtained from all participants of the research.

## Author contributions

NV: study conception, methodology, investigation, supervision, editing the manuscript. NM: methodology, material preparation, analysis, writing the first draft of the manuscript. KK: data curation, investigation, editing the manuscript. ED: software, analysis, visualization. All authors read and approved the final manuscript.
